# Insights into the SILAR Processing of Cu_*x*_Zn_1–*x*_S Thin Films via a Chemical, Structural, and Optoelectronic Assessment

**DOI:** 10.1021/acsomega.3c06848

**Published:** 2023-12-05

**Authors:** Dagoberto Cabrera-German, Miguel Martínez-Gil, Lorenzo Fuentes-Ríos, Zeuz Montiel-González, Dalia Alejandra Mazón-Montijo, Mérida Sotelo-Lerma

**Affiliations:** † Departamento de Investigación en Polímeros y Materiales, Universidad de Sonora, Blvd. Luis Encinas y Rosales s/n, Hermosillo, Sonora 83000, México; ‡ Departamento de Física, Matemáticas e Ingeniería, Universidad de Sonora, Campus Navojoa, Navojoa, Sonora 85880, México; § CONAHCYT-Centro de Investigación en Materiales Avanzados S. C., subsede Monterrey, Apodaca, Nuevo Leon 66628, México; ∥ Laboratorio de Diseño y Optimización de Recubrimientos Avanzados (DORA-Lab), CIMAV-Mty/TECNL-CIIT, Parque de Investigación e Innovación Tecnológica, Apodaca, Nuevo Leon 66629, México; ⊥ CONAHCYT-Tecnológico Nacional de México campus Nuevo León (TECNL), Centro de Investigación e Innovación Tecnológica (CIIT), Apodaca, Nuevo Leon 66629, México

## Abstract

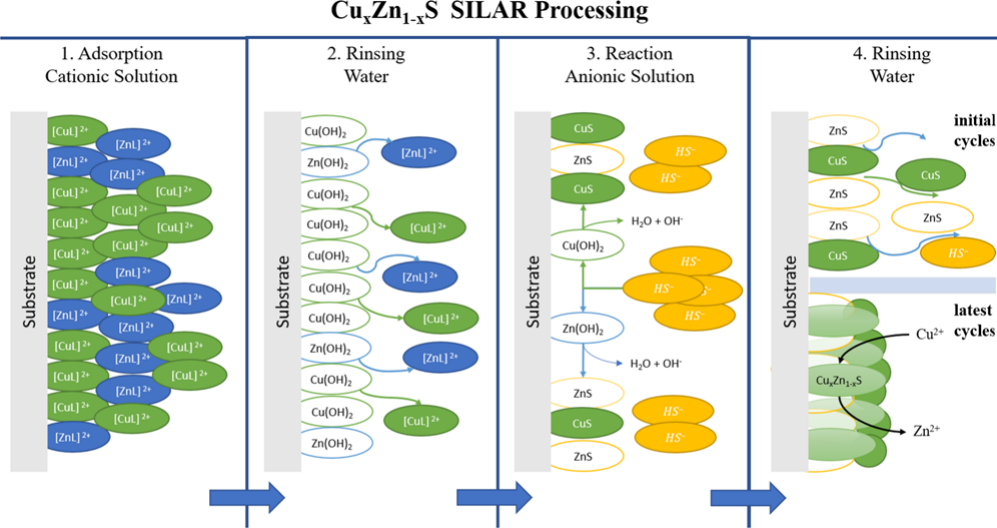

Careful analysis of the chemical state of Cu_*x*_Zn_1–*x*_S thin films remains an underdeveloped topic although it is key to a better understanding of the phase transformations and the linking between structural and optoelectronic properties needed for tuning the performance of Cu_*x*_Zn_1–*x*_S-based next-generation energy devices. Here, we propose a chemical formulation and formation mechanism, providing insights into the successive ionic layer adsorption and reaction (SILAR) processing of Cu_*x*_Zn_1–*x*_S, in which the copper concentration directly affects the behavior of the optoelectronic properties. Via chemical, optoelectronic, and structural characterization, including quantitative X-ray photoelectron spectroscopy, we determine that the Cu_*x*_Zn_1–*x*_S thin films at low copper concentration are composed of ZnS, metastable Cu_*x*_Zn_1–*x*_S, and CuS, where the evidence suggests that a depth compositional gradient exists, which contrasts with homogeneous films reported in the literature. The oxidation states for copper and sulfide species indicate that the films grow following a formation mechanism governed by ionic exchange and diffusion processes. At high copper concentrations, the Cu_*x*_Zn_1–*x*_S thin films are covellite CuS that grew on a ZnS seed layer. Hence, this work reiterates that future research related to fine-tuning the application of this material requires a careful analysis of the depth-profile compositional and structural characteristics that can enable high conductivity and transparency.

## 1 Introduction

Recent optoelectronic and photovoltaic technology challenges include the finding of the so-called suitable “p-type” transparent conductors (TCs), which are sought-after materials that must possess optical transparency and high electrical conductivity that can further improve the overall efficiency of several electronic devices.^1−4^ Furthermore, there is a keen interest in materials composed of abundant elements in the Earth’s crust, with low toxicity, and economically synthesized at low temperatures for applications in flexible transparent electronics.^3,5,6^

However, the latter characteristics are not easily met, and only a few materials have exhibited the potential to be used in optoelectronic and photovoltaic applications. For this purpose, a proven approach is the synthesis of ternary semiconductors by mixing or tuning the stoichiometry of binary systems, which in turn affect their optoelectronic properties. Notable TCs include nickel oxide (NiO), nickel–cobalt oxide (NiCo_2_O_4_), copper–aluminum oxide (CuAlO_2_), copper–chromium oxide (CuCrO_2_), and copper–iron oxide (CuFeO_4_). However, a rising interest in the development of nonoxide chalcogenide materials has occurred over the past few years^7,8^ due to the higher p-character compared to oxide-based TCs. In this way, as proposed by Mallik et al.,^3^ an attractive alternative is to synthesize nonoxide chalcogenide thin films of the Cu–Zn–S system, where it is intended to obtain Cu_*x*_Zn_1–*x*_S-type ternary compounds or (CuS)_*x*_/(ZnS)_1–x_-type nanostructured materials, where it is sought to have the transparency properties of ZnS and the conductive properties of CuS.^3,4^

Since its first report back in 2011, the ternary compound Cu_*x*_Zn_1–*x*_S has been synthesized in diverse forms by electrochemical deposition, pulsed laser deposition, spray pyrolysis, atomic layer deposition, and remarkably with low-temperature chemical solution methods, like, for example, chemical bath deposition and successive ionic layer adsorption and reaction (SILAR). This means that there exists an ongoing interest in developing high-quality Cu_*x*_Zn_1–*x*_S thin films due to their many versatile potential applications.^7−11^

There are still important aspects of the system that require further understanding regarding the chemical and structural nature. As accurately mentioned by Woods-Robinson et al.^4^ and shown throughout the literature, materials within the Cu–Zn–S system have characteristics that are strongly dependent on the synthesis method; essentially, the phase segregation of copper and zinc sulfide binaries, which determines whether a nanocomposite, a stable Cu_*x*_Zn_1–*x*_S ternary alloy, or a combination between the nanocomposite and the ternary alloy is obtained, further requires evidence for conclusive remarks.

In this study, we have focused on the SILAR deposition of Cu_*x*_Zn_1–*x*_S thin films because, to our knowledge, there has not been a detailed chemical state determination across the whole compositional range of this system. This aspect is worthy of addressing in greater detail because it may provide information about the stability of the thin films, their chemical state, and their sought-after optoelectronic properties. Moreover, in synthesizing the Cu_*x*_Zn_1–*x*_S compound via SILAR, there is still work to be done to confirm the mechanism that makes an n-type semiconductor, such as ZnS, turn into a high p-type conductivity obtained through the simple incorporation of copper.^4,12,13^ Therefore, the mechanism of incorporation of Cu into the films and its operation with the optoelectronic properties remain to be studied to produce films with potential application in transparent electronics.

Thus, in this work, we present further insights into the SILAR processing of thin films within the Cu–Zn–S system, hereby referred to as Cu_*x*_Zn_1–*x*_S thin films, as we have explored the chemical state and optoelectronic properties of the films upon varying the copper concentration in the SILAR cationic solution and studying the resulting synthesized thin films across a wide range (0 < *x* < 1) of Cu concentrations. We emphasize the chemical state of the films determined by state-of-the-art peak-fitting analysis of the photoelectron spectra that shows that as the covellite-like nature of the films increases, the films become semitransparent and their conductivity increases. This is important because the accurate chemical state determination contributes to a better understanding of the films, hence, leading to an improved application of the films in optoelectronic or photovoltaic devices. We demonstrate the feasibility of our proposed SILAR chemical formulation for the synthesis of Cu_*x*_Zn_1–*x*_S thin films and further propose a formation mechanism accounting for all the chemical species observed and demonstrating that the achieved optoelectronic properties can be modulated by choosing the adequate cation concentration.

Finally, the main novelties of the present work can be highlighted as follows.

1. Proposal of a novel SILAR-processing method featuring a unique chemical formulation. In contrast to existing reports where cation concentrations often exceed 0.1 M, our formulation employs significantly lower cation concentrations, typically not exceeding 0.005 M. This represents a reduction of up to 2 orders of magnitude in comparison to conventional methods (as reported elsewhere^1,14^). Additionally, our approach incorporates the use of sodium sulfide at reduced concentrations. This distinctive formulation not only offers a more cost-effective alternative but also contributes to environmental sustainability. By using less-concentrated reagents, we minimize the generation of concentrated residues, thus reducing the environmental footprint of the process. Furthermore, our method consistently yields high-quality semitransparent conductive thin films characterized by extensive coverage and exceptional reproducibility.
2. Use of advanced X-ray photoelectron spectroscopy (XPS) incorporating cutting-edge peak-fitting techniques,^15−18^ which allows to determine the chemical state of Cu_*x*_Zn_1–*x*_S thin films and provides precise estimations of their chemical composition. It is worth noting that our study adheres to the best practices in XPS analysis, avoiding common erroneous practices found in the literature, as highlighted in references.^19^,^20^ Our investigation delves into the intricate evolution of photoemission spectra, offering valuable insights into the complex electronic structure of this material.
3. Proposal of a formation mechanism tailored specifically to the SILAR process. The experimental data provides evidence suggesting an initial formation of a ZnS layer as a necessary first step in this mechanism. Subsequently, the incorporation of copper, facilitated by ionic exchange and diffusion interactions, occurs by letting the glass substrate be immersed in ionic solutions set at 65 °C for 10 s completing 50 SILAR cycles (the complete SILAR parameters are found in Table 1). The deposition was carried out in an air atmosphere. Our observed end points correspond to ZnS and covellite CuS, with the formation of a metastable Cu_*x*_Zn_1–*x*_S amorphous phase in between, where the three materials coexist in a compositional depth profile. This is an interesting aspect of the SILAR process. Our proposal sheds light on a critical challenge—how to synthesize high-quality CuS thin films directly on glass substrates. Until now, achieving this objective has been notably difficult without resorting to the use of seed layers like CdS^21^ or complex deposition techniques.

**Table 1:** SILAR Parameters for the Deposition of the Cu_*x*_Zn_1–*x*_S Thin Films

cationic solution			anionic solution			rinsing solutions		
*V*_Cu_	CuSO_4_	0.05 M	5 mL	Na_2_S	0.9 M	150 mL	distilled water	
where *V*_Cu_ = 1, 2, 3, 4, and 5 mL								
2.5 mL	ZnSO_4_	0.2 M						
1 mL	TEA	3.75 M						
1 mL	NH_4_OH	4.00 M	145 mL	distilled water				
145 mL—*V*_Cu_	distilled water							
cation stock solution concentration			0.33, 0.67, 1.00, 1.33, and 1.67 mM Cu^2+^; 3.33 mM Zn^2+^					
anion stock solution concentration			30 mM Na_2_S					

parameter	value
number of cycles	50
temperature of ionic solutions	65 °C
temperature of rinsing solutions	room temperature
immersion time for all solutions	10 s
immersion and retrieval speed	10 mm s^–1^
substrate transfer speed to the next step	10 mm s^–1^
time of a single SILAR cycle	65 s

## 2 Experimental Details

### 2.1 SILAR Deposition of Thin Films

Cu_*x*_Zn_1–*x*_S thin films were deposited by SILAR. The reagents employed for the preparation of the aqueous solutions were anhydrous copper(II) sulfate CuSO_4_ (98%) supplied by Spectrum; zinc sulfate heptahydrate ZnSO_4_·7H_2_O (99%) provided by Golden Bell reactivos; and ammonium hydroxide NH_4_OH (29% of NH_3_) acquired from Fermont. Additionally, the cationic solution enhanced the quality of the thin films by incorporating the ligand triethanolamine (TEA) N(CH_2_CH_2_OH)_3_ (99.8%) from Fermont. The anionic solution used sodium sulfide nonahydrate Na_2_S·9H_2_O (99.9%) from Fermont. Water with a resistivity of ∼500 kΩ was employed to prepare the stock reagent solutions and for rinsing as well. Fisherbrand Superfrost Plus Microscope Slides (25 × 75 × 1.0 mm^3^) without special preparation were used as substrates. These substrates were thoroughly washed with distilled water and Alconox detergent and subsequently rinsed with tap water, followed by distilled water and isopropyl alcohol. After the cleaning process, an immediate transfer to the SILAR deposition system was done.

The SILAR deposition of Cu_*x*_Zn_1–*x*_S thin films was carried out in a set of four 150 mL beakers (reactors). The first and third reactors contain the cationic and anionic solutions, respectively, while the second and fourth reactors contain the rinsing water. This study aimed to investigate Cu_*x*_Zn_1–*x*_S thin films prepared with SILAR by increasing the concentration of Cu^2+^ ions in a cationic solution. The cationic solutions were prepared by adding different volumes of a CuSO_4_ 0.05 M solution (*V*_Cu_) to a solution papered with 2.5 mL of ZnSO_4_ 0.2 M, 1 mL of TEA 3.75 M, and 1 mL of NH_4_OH 4.0 M. Finally, distilled water was added to obtain a final volume of 150 mL. A pH of ∼10 was measured using an MColorpHast indicator strip. The cationic solution was mixed at 65 °C and stirred for 1 min at 300 rpm with a magnetic stirrer. The *V*_Cu_ added during the preparation determines the concentration of Cu^2+^ ions in the cationic solution. For *V*_Cu_ of 1, 2, 3, 4, and 5 mL, the estimated concentrations in the cationic solution were 0.33, 0.67, 1.00, 1.33, and 1.67 mM CuSO_4_, respectively. The rinsing solutions consisted of 150 mL of distilled water. The anionic solution was prepared by adding 5 mL of 0.9 M Na_2_S to 145 mL of water; this solution was prepared and maintained at 65 °C. The pH obtained at the end was ∼10.

The SILAR depositions were conducted in a homemade system. It consists of a mechanical part of two rails coupled with stepper motors that allow movement in *x* and *z* coordinates, where the control of the stepper motors used A488 controllers and an Arduino Mega board, which also required the development of software using LabView. Each SILAR cycle was programmed with the parameters found in Table 1.

### 2.2 Characterization

With preliminary studies and while determining the best experimental conditions for a high-quality film, it was clear that, after 24 h of being exposed to the ambient atmosphere, the films are prone to oxidation at the surface. Therefore, to minimize the ambient oxidization, after deposition and between characterizations, the thin films were stored and transferred to the characterization tools using hermetic containers with 1.0 mbar pressure over the ambient and oxygen and humidity concentrations of 100 and 25 ppm, respectively.

The thickness and surface morphology of the films were studied with Zeiss Supra-40 scanning electron microscopy (SEM). Coupled with the SEM tool, an EDAX analyzer provided energy-dispersive spectroscopy data. UV–Vis–NIR transmittance and reflectance spectra were acquired using a Shimadzu UV–Vis–NIR 3600 spectrophotometer with the thin film facing the incident light and equipment calibration with air as the reference for transmission and a standard aluminum mirror for specular reflectance. X-ray diffraction (XRD) patterns were recorded employing a PANalytical Empyrean diffractometer operated under the grazing incidence configuration with a fixed omega angle of 0.5° and Cu Kα radiation (λ = 0.15406 nm) in the 2θ range from 20 to 90°. RAMAN spectroscopy was also employed to assess the structural characteristics of the films using a model Raman Thermo Scientific DXR spectrometer equipped with a 532 nm green laser. Electrical properties were measured with a Hall effect equipment Ecopia HSM 5000 model consisting of a four-point probe under a van der Pauw configuration. Finally, XPS was performed to assess the chemical nature of the films; the experiments were done using a SPECS PHOIBOS WAL analyzer and a monochromatic Al Kα_1_ (1486.7 eV) X-ray source, where the high-resolution spectra are obtained with a constant pass energy of 15 eV. The energy scale was referenced to the main adventitious C 1s peak centered at 284.8 eV. Detailed peak-fitting analysis was done using AAnalyzer software,^15^ where an important aspect of the analysis is the use of the active background approach.^16^

## 3 Results and Discussion

### 3.1 Thickness and Surface Morphology

**Figure 1:**
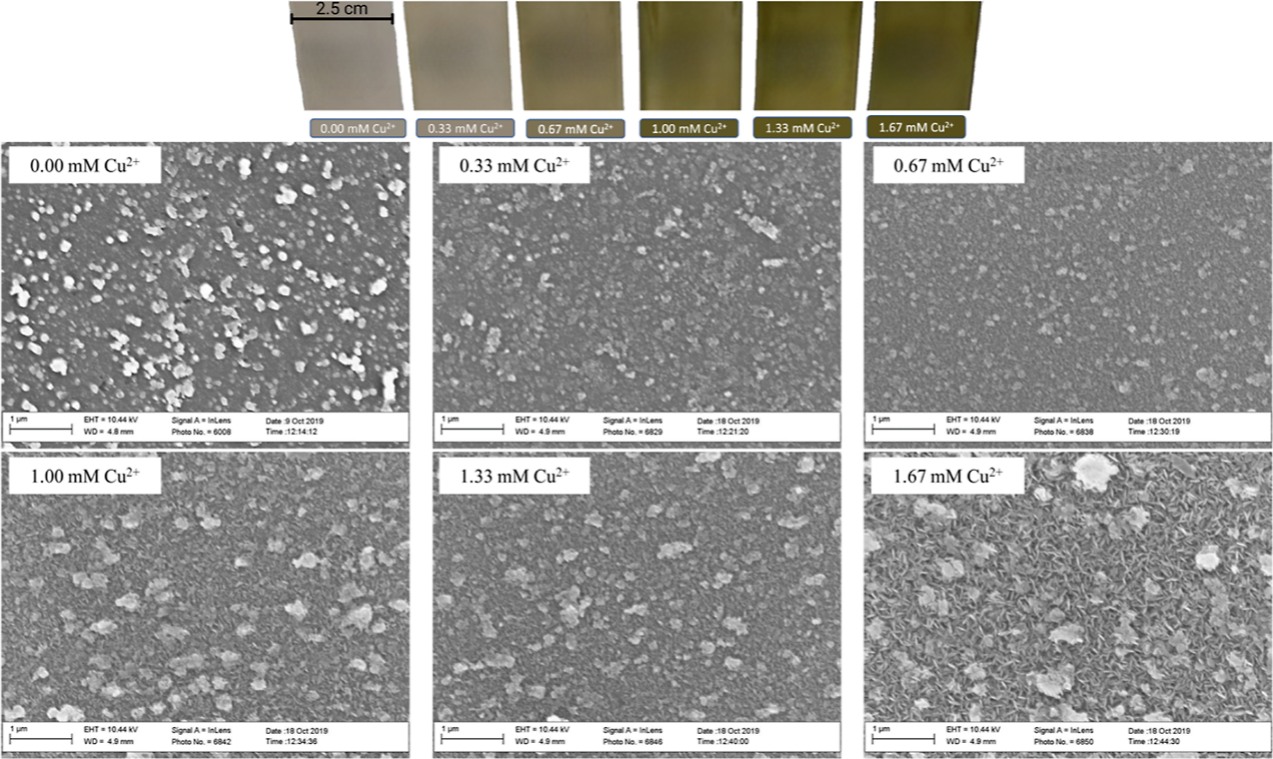
Top shows the appearance of the films after SILAR deposition, where a clear change in color is present with an increasing copper concentration in the cationic solution. The bottom section shows the evolution of the surface morphology of the Cu_*x*_Zn_1–*x*_S thin films with an increasing copper concentration in the cationic solution.

The resultant Cu_*x*_Zn_1–*x*_S thin films are found to be consistent and repeatable with the proposed SILAR methodology. In addition, the films adhere well to the substrate because even after a wet cotton swab is rubbed on the surface of the film, the glass substrate remains with material covering its surface. The appearance of the films can be appreciated in the top section of Figure 1, where it is possible to observe that the films turn from almost transparent for the ZnS film up to the characteristic green covellite color for the Cu 5 mL (1.67 mM Cu^2+^) thin film. The clear change in color is related to the incorporation of copper into the films caused by the increase in copper concentration in the cationic solution.

The surface morphology of the films also presents a clear evolution upon an increase in copper concentration in the cationic solution. It can be found in the bottom section of Figure 1 that first, the ZnS film exhibits a homogeneous and uniform surface morphology consisting of globular grains of around 300 nm. This morphology is characteristic of zincblende.^22,23^ Immediately after the cationic solution has copper ions, the surface morphology drastically changes, where the globular grains even present a decrease in size up to 100 nm in the case of the 1 mL of Cu (0.33 mM Cu^2+^) sample. By continuing the increase in copper concentration, the surface morphology keeps changing until incipient nanoplates emerge in the Cu 3 mL (1.00 mM Cu^2+^) sample. The nanoplates are like those reported for the microstructure of covellite thin films.^1,24,25^ The nanoplates are found to be homogeneously distributed across the film surface, having an aleatory orientation. After the clear change in surface morphology in the 1.00 mM Cu^2+^ sample, the nanoplate morphology does not appear to change with increasing copper concentration in the cationic solution, where only the nanoplates appear to be more clearly defined and increasing in size reaching about 300 nm longitudinally for the case of the Cu 5 mL (1.67 mM Cu^2+^) sample.

**Figure 2:**
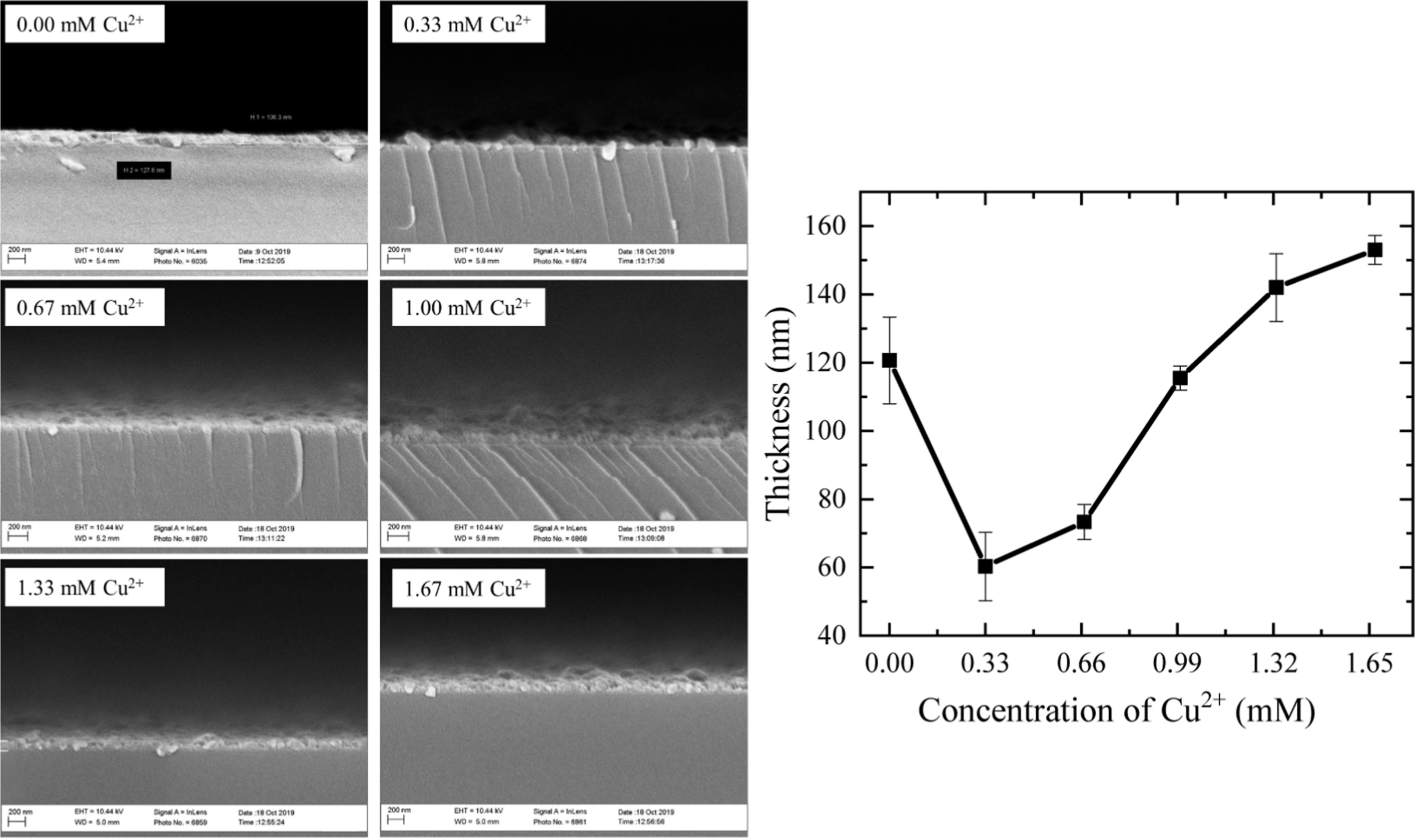
Thickness of the Cu_*x*_Zn_1–*x*_S thin films with increasing copper concentration in the cationic solution is shown by cross-sectional SEM images.

Figure 2 (left side) shows the cross sections of the films, where the film thickness also presents a dramatic reduction going from the ZnS to the Cu 1 mL (0.33 mM Cu^2+^) sample, and from 0.33 mM Cu^2+^ up to 1.67 mM Cu^2+^, the films increase in thickness almost linearly, as expected from the SILAR method. It is possible to observe that overall, the films indeed have uniformity across the surface of the glass substrate forming conformal thin films, with the only exception being the 0.33 mM Cu^2+^ sample, where the integrity of the film appears to be compromised as the film is far less uniform when compared to the other films. The trend in thin film thickness with different concentrations of copper in the cationic solution is presented in Figure 2 (right side). The thickness of the films is also drastically affected by the introduction of copper ions into the cationic solution, but after that, with increasing copper concentration, the film thickness increases almost in a linear fashion.

These results may help to formulate a possible formation mechanism of the Cu_*x*_Zn_1–*x*_S thin films, consisting of the formation of a possible ZnS film that serves as a seed layer for copper ions to slowly incorporate via a cationic exchange, possibly up to the formation of a copper sulfide seed film, since the thicker films exhibit the typical covellite nanoplate surface morphology of this material.

### 3.2 UV–Vis–NIR Transmittance and Reflectance Spectra

**Figure 3:**
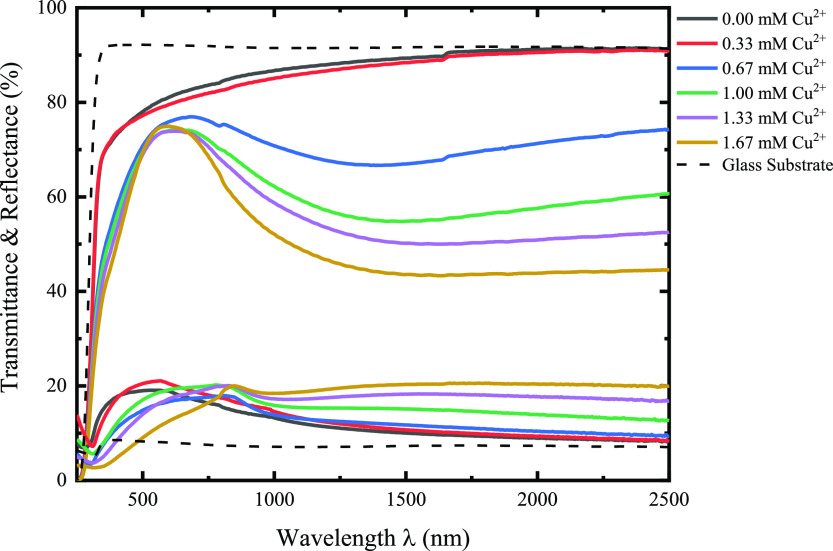
Transmittance and reflectance spectra of Cu_*x*_Zn_1–*x*_S thin films with an increasing copper concentration in the cationic solution.

The results of the UV–Vis–NIR characterization are listed in Figure 3. It can be observed that the color change, influenced by the increase in copper concentration in the cationic solution, is related to the decrease in transmittance in the visible region. Moreover, the edge of the fundamental transition, between 300 and 500 nm, is shifted to longer wavelengths with an increasing copper concentration in the cationic solution, which explains the transition in color from transparent for the ZnS sample to green covellite of the 1.67 mM Cu^2+^ sample.

It is interesting to observe a sharp shoulder around 600 nm, which is a typical characteristic of covellite CuS, according to several reports.^22,26−28^ It can be appreciated that the transparency at 600 nm is the highest for the 0.00 mM Cu^2+^ sample, reaching around 80%, where the composition of this film corresponds completely to the ZnS thin film. As the Cu content increases, the transparency in the visible region decreases, but the sharp shoulder is still not present, a behavior previously seen in (CuS)_0.17_/(ZnS)_0.83_ thin films,^28^ which according to our chemical composition results for the 0.33 mM Cu^2+^ sample, yielding Cu_0.08_Zn_0.92_S_1.03_ (the complete chemical composition analysis is presented in Table 2), this spectrum might be related to the presence of this ternary compound in the films. The transmittance spectrum for the 0.67 mM Cu^2+^ sample in contrast now exhibits the sharp shoulder but with higher transparency when compared to the remaining samples that are in complete correspondence to the expected CuS covellite spectrum.^22,26−28^ Here, at a chemical composition of Cu_0.17_Zn_0.83_S_1.02_ for the 0.67 mM Cu^2+^ sample, the averaged transmittance spectrum is obtained between both the metastable Cu_*x*_Zn_1–*x*_S amorphous phase and the incipient CuS covellite phase, in agreement with the XRD and XPS analysis.

**Table 2:** Atomic Composition of the Cu_*x*_Zn_1–*x*_S Thin Films with Increasing Copper Concentration in the Cationic Solution Was Estimated by Photoelectron Spectroscopy^a^

		atomic percentage %										
		Zn 2p		Cu 2p			S 2p			O 1s	Cu_*x*_Zn_1–*x*_S_*y*_	
sample ID	Cu/Zn ratio in the cationic solution	Zn–S	Zn–O	Cu_1_	Cu_2_	S_1_	S_2_	S_d_	S_d′_	O_met_	XPS	EDS
0.00 mM Cu^2+^	0.00	43.6	5.9	0.0	0.0	44.0	0.0	0.0	0.0	6.5	Zn_1.00_S_1.01_	Zn_1.00_S_0.92_
0.33 mM Cu^2+^	0.10	13.0	8.8	13.0	7.4	2.7	32.4	11.6	6.8	4.4	Cu_0.61_Zn_0.39_S_1.39_	Cu_0.08_Zn_0.92_S_1.03_
0.67 mM Cu^2+^	0.20	14.5	2.5	13.6	9.3	11.6	22.0	10.7	10.9	4.9	Cu_0.61_Zn_0.39_S_1.19_	Cu_0.17_Zn_0.83_S_1.02_
1.00 mM Cu^2+^	0.30	9.4	2.2	16.9	11.9	13.1	26.5	12.1	2.7	5.2	Cu_0.75_Zn_0.25_S_1.36_	Cu_0.32_Zn_0.68_S_0.97_
1.33 mM Cu^2+^	0.40	7.8	1.8	18.1	13.8	13.0	25.2	9.6	5.4	5.3	Cu_0.80_Zn_0.20_S_1.20_	Cu_0.49_Zn_0.51_S_1.01_
1.67 mM Cu^2+^	0.50	2.6	1.7	23.4	16.8	10.4	23.9	11.7	3.7	5.8	Cu_0.94_Zn_0.06_S_1.08_	Cu_0.74_Zn_0.26_S_1.03_

a For comparison, an estimation with EDS is also included.

Additionally, the decrease in transmittance is influenced by the increasing film thickness for samples having copper because copper sulfide absorbs more light in the visible and near-infrared regions. The monotonic decrease of the transmittance in the NIR region is evidence suggesting that the films have an increasing metallic character as it has been reported for CuS.^8,23,28,29^

The reflectance spectra also show notable changes, as we can see for the 0.33 mM Cu^2+^ sample that the possible increase in copper in the films significantly increases the reflective characteristics. By comparing ZnS with 0.33 mM Cu^2+^, even though ZnS is approximately 70 nm thicker, the 0.33 mM Cu^2+^ sample reflects more light over the whole electromagnetic region measured. With the incorporation of copper as the copper concentration in the cationic solution increases, the reflectance in the NIR region increases, revealing again the metallic character of the CuS films through the appearance of the typical behavior of an absorption edge due to the plasma resonances of free charge carriers. It is possible to observe large and wide shoulders, which might be an indication of the typical response of a predominantly amorphous character (wide reflection bands) that remains until the 1.67 mM Cu^2+^ sample, which tends to crystallize (sharp reflection bands) as implied by the well-defined maximum in the reflectance spectrum between 750 and 1000 nm, which is in accordance with the microstructure observed in the surface morphology of the films.

**Figure 4:**
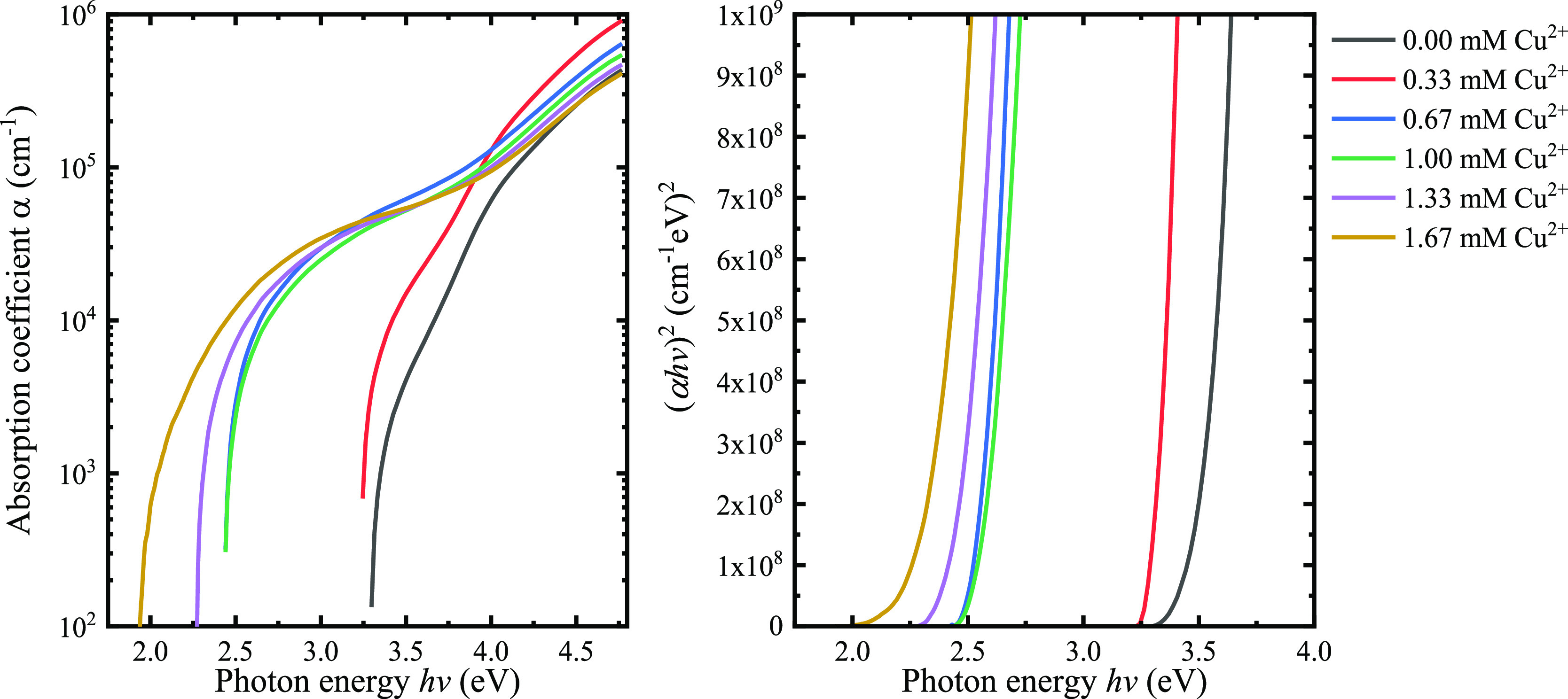
Left: absorption coefficient of Cu_*x*_Zn_1–*x*_S thin films. Right: energy bandgap determination via Tauc plot considering direct transitions.

The absorption coefficient (α) determination assumed that the internal reflection model applies to the present thin films following a previously reported methodology.^30,31^ The absorption coefficient for all samples is presented in Figure 4 (left), where two clear pronounced shoulders are notable, located around 2.5 and 3.8 eV, which may be attributed to the absorption edges corresponding to covellite and ZnS,^3,25,29^ respectively. It can be noted that the absorption coefficient has a complex behavior, which is the result of the presence of at least three different bandgap materials (glass substrate, ZnS, and CuS), despite those differences in light scattering at the surface of the films caused by the varying surface morphology as the copper concentration in the cationic solution increases, could contribute to this complexity as well.

The bandgap energy (*E*_g_) for each Cu_*x*_Zn_1–*x*_S thin film was determined using the Tauc method assuming direct transitions with results shown in Figure 4 (right). The *E*_g_ value for the ZnS sample is found to be 3.5 eV, corresponding to ZnS,^22,25^ and with an increasing copper concentration in the cationic solution, the E_g_ decreases up to 2.37 eV for the 1.67 mM Cu^2+^ sample, the value explained by the direct energy band gap of CuS covellite.^21,22,28^ Interestingly, we can observe that the 0.67 mM Cu^2+^ sample slightly falls out of the trend that the other samples follow, and the sudden decrease in the *E*_g_ may be due to a possible formation of a transition phase that forms between the ZnS and CuS covellite end points.

### 3.3 Structural Analysis

**Figure 5:**
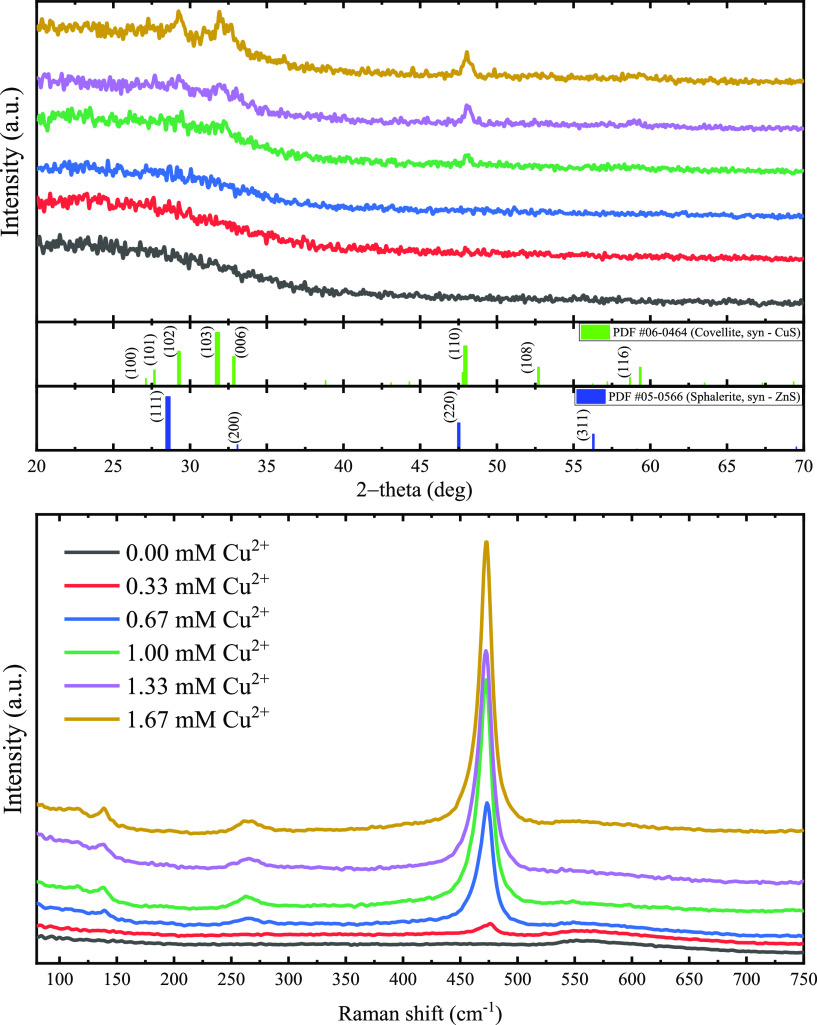
XRD patterns and Raman spectra of the Cu_*x*_Zn_1–*x*_S thin films with increasing copper concentration in the cationic solution.

The structural characteristics of the films were studied first with XRD, and the patterns are found in the top section of Figure 5 where at first glance, the low-intensity diffraction peaks indicate that the films are predominantly amorphous. However, as was seen in the surface morphology analysis where, with an increasing copper concentration in the cationic solution, the characteristic nanoplates of CuS covellite emerge and grow, in the diffraction patterns starting at the 1.33 and 1.67 mM Cu^2+^ samples, low-intensity diffraction peaks are seen, all of them being in complete correspondence to the indexed PDF #06-0464 of CuS covellite. This implies that the Cu_*x*_Zn_1–*x*_S thin films grow from an amorphous ZnS film that gradually transforms into films crystallizing in the CuS covellite structure (*P*6_3_/*mmc*).

For a better understanding of the structural characteristics and because the contribution of the glass substrate to the XRD patterns might hide certain features, we also measured the Raman spectra of the Cu_*x*_Zn_1–*x*_S thin films. The results are presented in the bottom section of Figure 5 with sharp Raman peaks appearing at 474, 265, and 140 cm^–1^ that are also in correspondence with the CuS covellite phase.^24,32^ The sharp peak located at 474 cm^–1^ exhibits a clear dependence on the increase of the copper concentration in the cationic solution, starting from an asymmetric low-intensity peak for the 0.33 mM Cu^2+^ sample up to a sharp symmetric large-intensity Raman peak found for the 1.67 mM Cu^2+^ sample. The asymmetry at low concentrations of copper may indicate resultant phases such as that of the CuS covellite structure with low order or strained that with increasing copper concentration gradually crystallizes to a well-defined CuS covellite phase. Here, we note that the broad asymmetric peak found for the 0.33 mM Cu^2+^ sample has a maximum of around 477 cm^–1^, which slightly shifts to a lower Raman frequency of 475 cm^–1^ for the Cu 0.67 mM Cu^2+^ sample and 474 cm^–1^ for the rest of the samples, which might imply that for the samples, prepared with a low concentration of copper in the cationic solution, the Cu_*x*_Zn_1–*x*_S thin films have considerable induced stress.^24^ No other additional phases were detected, apart from the band around 550 cm^–1^ corresponding to second-order vibrational modes of the cubic β-ZnS phase^22^ that appears to be predominantly amorphous due to the low scattering intensity.

### 3.4 Electrical Properties

**Figure 6:**
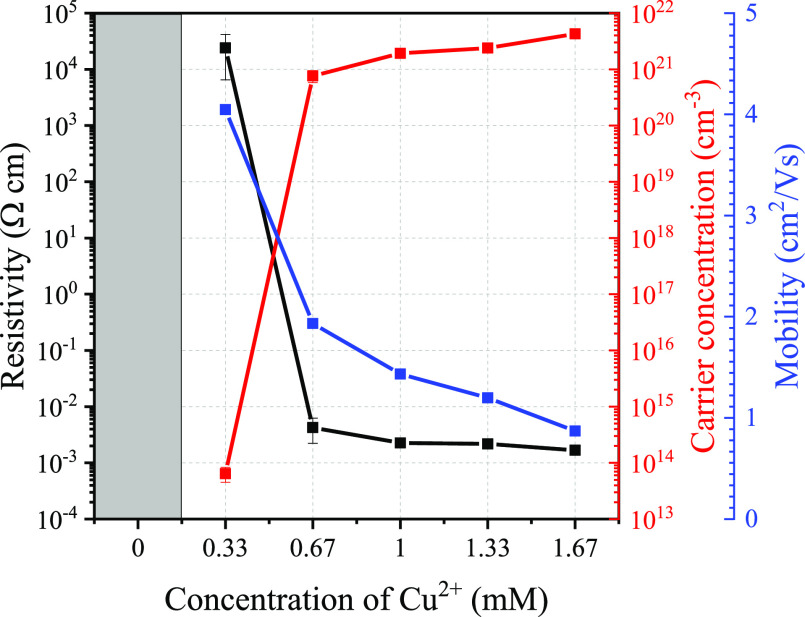
Electrical properties of the Cu_*x*_Zn_1–*x*_S thin films. The gray area indicates that the measurement of the sample was not possible due to the high resistivity of the film.

The determination of the electrical properties of the Cu_*x*_Zn_1–*x*_S thin films is presented in Figure 6. First, for the reference ZnS sample, under the present experimental conditions, no reliable data could be obtained because the resistivity was too large. However, upon incorporation of copper into the films, the electrical measurements were found to be reliable. For all the remaining samples, the conductivity was found to be p-type according to the measured Hall coefficient and these results are in correspondence with other reports found in the literature.^2,3,22,28,33^ It is possible to observe that the carrier concentration increases with an increasing copper concentration in the films, going from 10^13^ up to 10^21^ cm^–3^ spanning almost 8 orders of magnitude in the studied range. From the behavior of the carrier concentration, an increase of the copper concentration in the cationic solution will not improve the electrical properties because we have reached the maximum that corresponds to covellite deposited by a SILAR methodology.

The carrier mobility decreases with an increasing copper concentration in the thin films, decreasing from 4 and reaching 1 cm^2^/(V s), where these values are like others reported elsewhere.^1,28^ This decreasing mobility trend may be influenced by the continuous formation of tiny CuS covellite crystals in the films, which increase the carrier concentration but decrease the mobility simultaneously. The decrease in mobility is therefore mainly attributed to the varying morphology of the films that affect the transport of holes through the films, where the increasing incipient crystallites act as trapping centers hindering the carrier mobility.^34,35^

### 3.5 Chemical State Assessment

**Figure 7:**
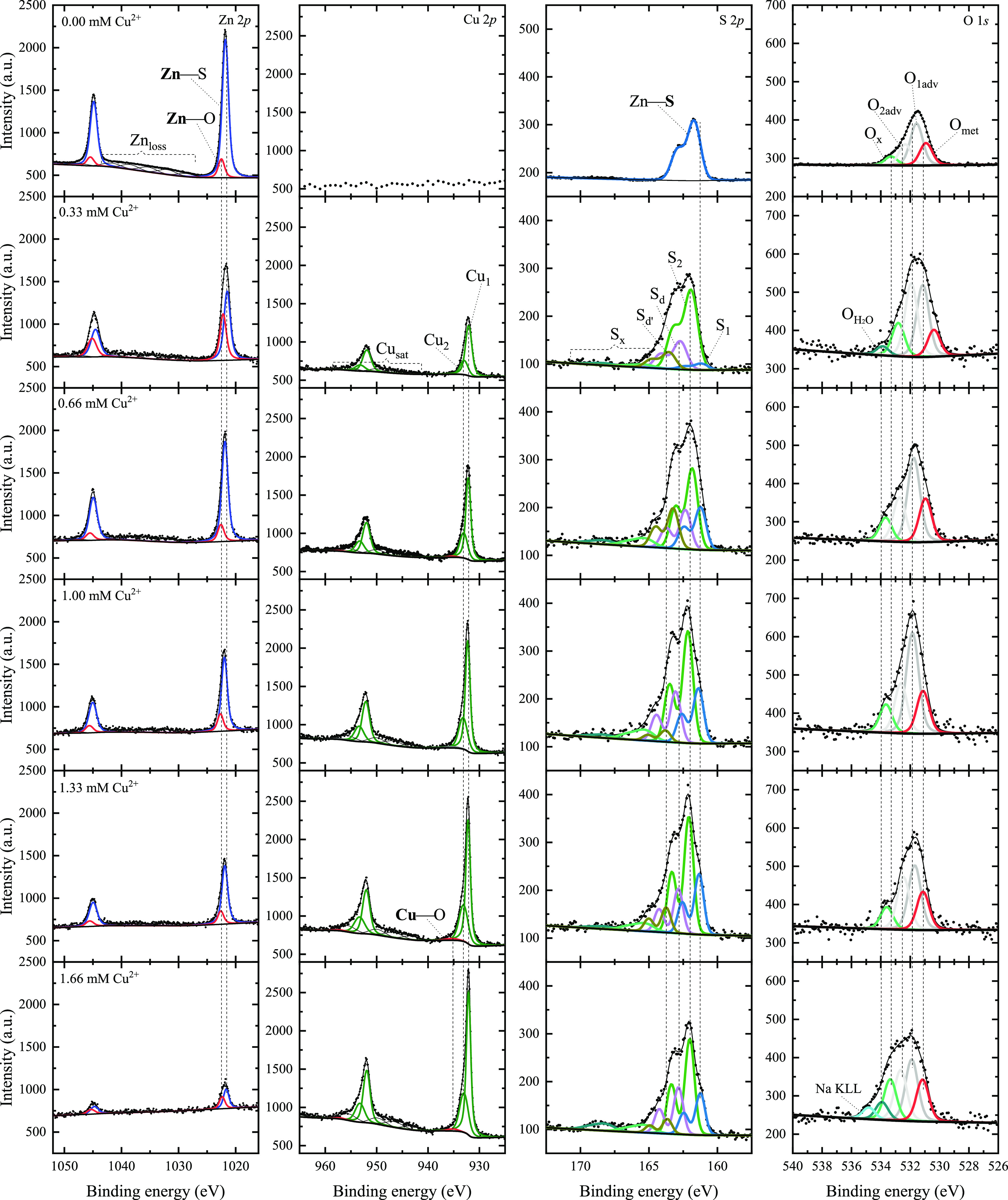
X-ray photoelectron spectra and peak-fitting analysis of Cu_*x*_Zn_1–*x*_S thin films with an increasing copper concentration in the cationic solution. A clear intensity evolution is observed according to the chemical state that the Cu_*x*_Zn_1–*x*_S thin films have with different copper concentrations.

Up to this point, the previous characterization suggests that the presence of copper ions in the cationic solution indeed incorporates copper atoms in the Cu_*x*_Zn_1–*x*_S thin films. To assess the resultant composition and establish the relationship between the copper ion concentration in the cationic solution and the copper content in the resultant films, we have quantitatively analyzed the high-resolution photoelectron spectra of the films. A detailed description of the peak-fitting methodology can be found elsewhere.^16,36,37^ The detailed peak-fitting analysis is shown in Figure 7. Overall, by visual inspection of the Cu 2p and the Zn 2p spectra, we observe contrasting behaviors of the intensity of the main photoemission peaks with increasing copper concentration in the cationic solution; we get that the Zn 2p total signal decreases, and the Cu 2p total signal increases. This result is direct evidence of the presence of copper ions being incorporated into the Cu_*x*_Zn_1–*x*_S thin films, as corroborated by the complementary characterization present in the previous sections of this work. Therefore, by simply choosing the copper ion concentration in the cationic solution, it is possible to tailor the final copper content in the resultant thin film via the SILAR methodology proposed here.

For further detail, the Zn 2p core level shows that the main peak is composed of two signals, one energy located on average at 1021.70 ± 0.35 eV binding energy corresponding to zinc atoms bonded to sulfur (labeled as Zn–S) and another centered at 1022.40 ± 0.35 eV binding energy related to zinc oxide species (labeled as Zn–O).^38^ There is also the presence of complex peaks located in the region of 1025–1045 eV that are related to loss features arising from scattering events that photoelectrons experience in their travel out of the solid. By comparing the 0.00 mM Cu^2+^ (1021.76 ± 0.05 eV) and 0.33 mM Cu^2+^ (1021.46 ± 0.08 eV) samples, the results show that the binding energy of the peak related to ZnS shifts 0.3 eV to lower binding energies just as copper ions are present in the cationic solution. As the copper concentration in the cationic solution increases, the resultant films show a shift to lower binding energies lying within 0.2 eV, which still may indicate that the local chemistry of the ZnS is affected by the incorporation of the copper ions, meaning that the resultant thin films are not a straightforward nanostructured material, and the shift in binding energies with respect to the position of the ZnS sample suggests that the original pure Zn–S bonding is affected by the formation of possible Zn–S–Cu bonds within the Cu_*x*_Zn_1–*x*_S thin films.

The Cu 2p photoemission spectra do not seem to have significant changes in terms of energy shifts, meaning that with an increasing copper concentration in the cationic solution, the energy position does not vary, only the intensity changes. We observe that the spectra are mainly composed of two peaks. First, labeled as peak Cu_1_ at 932.12 ± 0.10 eV binding energy, we have a signal that is in correspondence to the Cu^+^ state of the CuS covellite phase.^26,36,39−42^ Also, as reported in other reports on the photoemission spectra of CuS covellite, there is the presence of a peak centered at 933.02 ± 0.15 eV (peak Cu_2_), that in the present case may very well be more suited to a Cu^+^ state coming from copper sulfides with symmetries slightly different to that of the CuS covellite, possibly coming from induced stress of the Cu_*x*_Zn_1–*x*_S lattice around the neighborhood of the Cu atoms. Furthermore, the peak can be explained by a mixed ground-state configuration also characteristic of a Cu^+^ state.^40,43−45^ Nevertheless, the contribution of a Cu^2+^ state cannot be neglected as well.^26,46^ The Cu 2p spectra also have satellite features in the 943–950 eV binding energy region, which are features also found in other copper sulfides^26,36^ coming from loss peaks, and peaks related to ground-state configurations intrinsic to the Cu^+^ and Cu^2+^ photoemission spectra. It is interesting to note that, despite the precursor employed being a Cu^2+^ salt, the resultant films show that the copper ions during the thin film processing are reduced to at least Cu^+^ to a significant extent. Regardless of accurately attributing the nature of all of the peaks found in the Cu 2p spectra, we can say that peaks Cu_1_ and Cu_2_ indeed come from copper sulfide species. We can observe a minority peak, labeled as Cu–O at 935.20 ± 0.15 eV binding energy, that accounts for the atmospheric formation of copper oxides at the covellite-type surface.^41^

Regarding the S 2p photoemission spectra, they exhibit a strong difference across all samples. First, for the 0.00 mM Cu^2+^ (ZnS) sample, the spectrum is straightforward consisting of a sole doublet peak located at 161.69 ± 0.06 eV directly related to the ZnS compound, the reason for its labeling Zn–S as for sulfides bonded to zinc. However, after the initial incorporation of copper into the films, the S 2p suffers a complex evolution up to the 1.67 mM Cu^2+^ sample, being the one with the highest content of copper, as shown by the high intensity of the Cu 2p spectrum and the low intensity of Zn 2p. When the atomic content of copper in the Cu_*x*_Zn_1–*x*_S thin films is the highest (1.67 mM Cu^2+^ sample), the photoemission peaks can be directly ascribed to the sulfide peaks found in CuS covellite, where the peak centered at 161.23 ± 0.05 eV in the lower-binding energy side of the spectrum and labeled as S_1_ corresponds to an S^2–^ chemical state.^39,40,47−49^ However, as the copper content in the films is varied, the S_1_ peak does not show a consistent position; meaning that while for the 0.33 mM Cu^2+^ sample, the peak is shifted down to a 161.15 ± 0.55 eV binding energy when compared to the original 161.69 ± 0.06 eV position corresponding to 0.00 mM Cu^2+^, in turn, for the 1.00 mM Cu^2+^ film, the energy position shifts back to 161.37 ± 0.08 eV binding energy. These results might indicate that the chemical nature of the S^2–^ state changes with increasing copper concentration in the Cu_*x*_Zn_1–*x*_S thin films; likely as copper is incorporated into ZnS, the lattice is strained or modified with the formation of structural defects or Zn–S–Cu bonds, respectively, that could be arising from a kind of ionic exchange mechanism.

The latter description is similar for peaks S_2_ and S_d_ that, for the 1.67 mM Cu^2+^ sample, they are located at 162.00 ± 0.05 and 162.82 ± 0.09 eV binding energy with a nature related to a disulfide (S_2_)^2–^ state^39,40,47−49^ and nonstoichiometric sulfides (S_*x*_)^2–^, respectively.^26,36,50^ Here, the chemical shifts are not so pronounced, and assigning statistical significance is difficult due to the several overlapping peaks comprised in the spectra, but shifts are still present with increasing copper content in the Cu_*x*_Zn_1–*x*_S thin films. We observe the rise of the S_2_ peak for the 0.33 mM Cu^2+^ film centered at 161.91 ± 0.30 eV, reaching a minimum for the 0.67 mM Cu^2+^ film at 161.80 ± 0.20 eV and a maximum in the 1.00 mM Cu^2+^ film at 162.14 ± 0.08 eV binding energy. Also, there exists the presence of the additional S_d′_ peak related to nonstoichiometric sulfides (S_*x*_)^2–^ coming from undetermined copper sulfide phases or possible oxoanions of sulfur from byproducts of the SILAR deposition, which is energy located at 163.63 ± 0.18 eV binding energy in the case of the 1.67 mM Cu^2+^ film. We note that peak S_d′_ has almost the same position for all samples within a ±0.10 eV range, except for the 0.67 mM Cu^2+^ film that shows a clear downshift to 163.22 ± 0.22 eV binding energy.

As previously noted, the photoelectron spectral characteristics of the CuS covellite are complex, and these appear to be further complicated in the in-between transition from the simple S 2p spectrum of ZnS and the spectrum of CuS covellite in the 1.67 mM Cu^2+^ sample. The presence of various peaks alongside their unique shifts and very particular relative intensities (see Table 2 for peak intensity quantitation) indicates the possible formation of metastable ternary structures. However, due to the complexity of the photoemission peaks, the presence of polymorphs or even the formation of Zn–S–Cu crystalline structures is still possible as suggested by Woods-Robinson et al.^4^ Hence, our results suggest the formation of a metastable Cu_*x*_Zn_1–*x*_S structure with sulfide chemical states that remain to be accurately ascribed to a certain sulfide bonding or electronic configuration adequate to the crystal field of the ternary structure. It could undoubtedly be inferred that the ZnS thin film, corresponding to the 0.00 mM Cu^2+^ sample and the one mainly composed of the CuS covellite 1.67 mM Cu^2+^ film, possesses two completely different electronic structures as shown by the shape, binding energy positions, and relative intensity of the photoemission signals. Hence, the same can be said for the in-between films that show photoemission signals and thus electronic structures that differ from the pure ZnS and CuS covellite, giving the possibility of the existence of Cu_*x*_Zn_1–*x*_S lattices or at least Zn–S–Cu bonding given the resultant varying photoemission spectra.

The remaining peaks in the S 2p photoelectron spectra are collectively labeled as S_*x*_, and they are noteworthy found to lie at the same binding energy position for all samples, mainly located at 165.25 ± 0.20 and 168.15 ± 0.08 eV. The presence of these peaks can be mainly attributed to the presence of elemental sulfur,^51,52^ whose presence in the films can be explained by the formation mechanism presented later, and the oxoanions of sulfur, predominantly sulfates,^53,54^ which are the residue of the precursors employed in the SILAR processing.

The O 1s photoemission spectra also show interesting results. It is observed that even with careful handling of the films, surface oxidation is inevitable because ZnO is more stable than both ZnS and CuS covellite, and when exposed to atmospheric pressure, the films tend to form a copper oxide layer at the surface,^36,41^ all of which explain the presence of several oxygen features in the O 1s spectra. The photoemission signal located around 531.5 eV may be related to metallic oxides such as ZnO and CuO; however, its out-of-range location from the expected 529.5–530.5 eV interval^37,55,56^ further indicates that these metallic oxides are amorphous or maybe they are nonstoichiometric. The additional peaks might as well have several origins, mainly organic compounds adhered to the surface by exposure to the atmosphere^57−59^ or byproducts of the SILAR reactions such as sulfates, sulfites, or sulfur suboxides,^27,60−62^ as expected because the films were synthesized at atmospheric pressure. In this case, these are assumed to be surface components that do not inherently belong to the Cu_*x*_Zn_1–*x*_S films.

Another focus was the accurate estimation of the chemical composition of the Cu_*x*_Zn_1–*x*_S thin films. Our approach has been previously used with success in the chemical assessment of other copper sulfide thin films with further details found elsewhere.^26,36^ The results are presented in Table 2, where the atomic percentage was calculated by considering the attenuation of the photoemission signal due to scattering and assuming a homogeneous film across the depth sensibility of the XPS technique. Here, the assessed intensity of the photoemission peaks was corrected using physical parameters appropriate for each photoelectron signal.^36,56,63^ The results clearly show what was observed in the photoelectron spectra presented in Figure 7, that is, the decrease in intensity of the Zn 2p signals and the intensity increase of the Cu 2p peaks as the copper concentration in the cationic solution increases. With this, we clearly show that copper is incorporated into the Cu_*x*_Zn_1–*x*_S thin films, where copper is found to be easily diffused into the film because even at Cu/Zn ratios in the cationic solution below 0.50, we obtain a large amount of copper atoms in the films. We also notice that the intensity of the peaks present in the S 2p does not show a clear trend with varying copper concentrations, hence showing varying atomic percentages across the changing copper content in the films. These results again demonstrate the complexity of the surface chemistry of the films.

The chemical composition of the Cu_*x*_Zn_1–*x*_S thin films was estimated by determining the value for *x* and *y* and choosing the photoelectron signals that account for the sulfides. For example, the *x* ratio was quantified with the corresponding signals that are bonded to sulfur and pertain only to the Cu_*x*_Zn_1–*x*_S film: *x* = Cu_Tot_/(Cu_Tot_ + Zn_Tot_), where Cu_Tot_ = Cu_1_ + Cu_2_, both being part of copper sulfide and Zn_Tot_ = Zn–S, the sole peak in the Zn 2p spectra related to a metal sulfide. The *y* ratio was determined using the relationship *y* = S_Tot_/(Cu_Tot_ + Zn_Tot_), where S_Tot_ equates to the total intensity of the sulfur species related to the Cu_*x*_Zn_1–*x*_S film, that is, S_Tot_ = S_1_ + S_2_ + S_d_. The resultant compositions demonstrate that at the surface of the films, the copper content is significant because at low cation concentrations such as in samples 0.33 and 0.67 mM Cu^2+^, the synthesized films have up to an *x* = 0.61 of copper concentration, already a Cu/Zn ratio that surpasses 1.5:1. After the 1.00 mM Cu^2+^ sample, the copper content gradually increases reaching *x* = 0.94 of copper concentration. We also obtain that the amount of sulfur in the films is relatively high, reaching a maximum of *y* = 1.39 for the 0.33 mM Cu^2+^ sample and on average exhibiting a *y* = 1.24 sulfur composition. These composition results support that it is possible that the hypothetical ternary Cu_2_ZnS_2_ phase or a Cu_2_ZnS_2_ polymorph could have been obtained during film deposition and they can be present in samples up to 0.67 mM Cu^2+^. Although we were not able to produce direct structural evidence and the sulfur content is relatively large deviating from the *y* = 0.66 suggested by Woods-Robinson et al.,^4^ the composition and photoelectron spectra can suggest that the ternary phase is accessible, and further detailed studies are required.

For comparison purposes, we also estimated composition via energy-dispersive spectroscopy (EDS) measurements with contrasting results. However, the results cannot be accounted for in full because of the large uncertainties related to the total content of zinc and copper, where it is not possible to discriminate between sulfide and oxide species. The results still show that the films do not have a uniform composition across the depths of the films. The strong differences in sampling depth between the two techniques employed indicate that there is a copper composition gradient. This suggests that, in the region near to the substrate, the copper content is scarce, and as we get close to the surface region that is exposed to the cationic and reaction solution, the copper content is more predominant. The latter could also hint that the formation mechanism of the Cu_*x*_Zn_1–*x*_S thin films is driven by ionic exchange and diffusion interactions. Also, from the 1.00 mM Cu^2+^ sample, we can say that the films, at least at their surface, are predominantly CuS covellite with Zn doping, while in the near-substrate region, it is the other way around with ZnS having a degree of Cu doping.

### 3.6 Formation Mechanism

Through the characterization presented in the previous sections of this work, it is possible to elaborate a formation mechanism that accounts for the structural, electrical, and chemical properties of the thin films with increasing incorporation of copper. The mechanism proposed is based on the well-known metallurgical copper activation mechanism of the sphalerite surface.^33,64−70^

The formation mechanism consists of a series of concatenated steps. In the preparation of the cationic solution, metallic ion sources dissociate in aqueous media, forming aqueous ions as follows
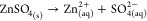
1
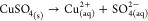
2

As mentioned in the Experimental Details Section, the cationic solution also employed ligands to enable a controlled presence of free ions in the vicinity of the glass substrate. The ammonium and TEA ligands also provide a basic medium by releasing OH^–^ via their dissociation in the aqueous medium, making the cationic solution have a pH of ∼10. Therefore, the species present in the cationic solution may be described by the following equilibrium reactions^71−73^
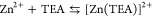
3
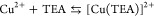
4
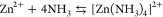
5
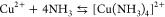
6

**Figure 8:**
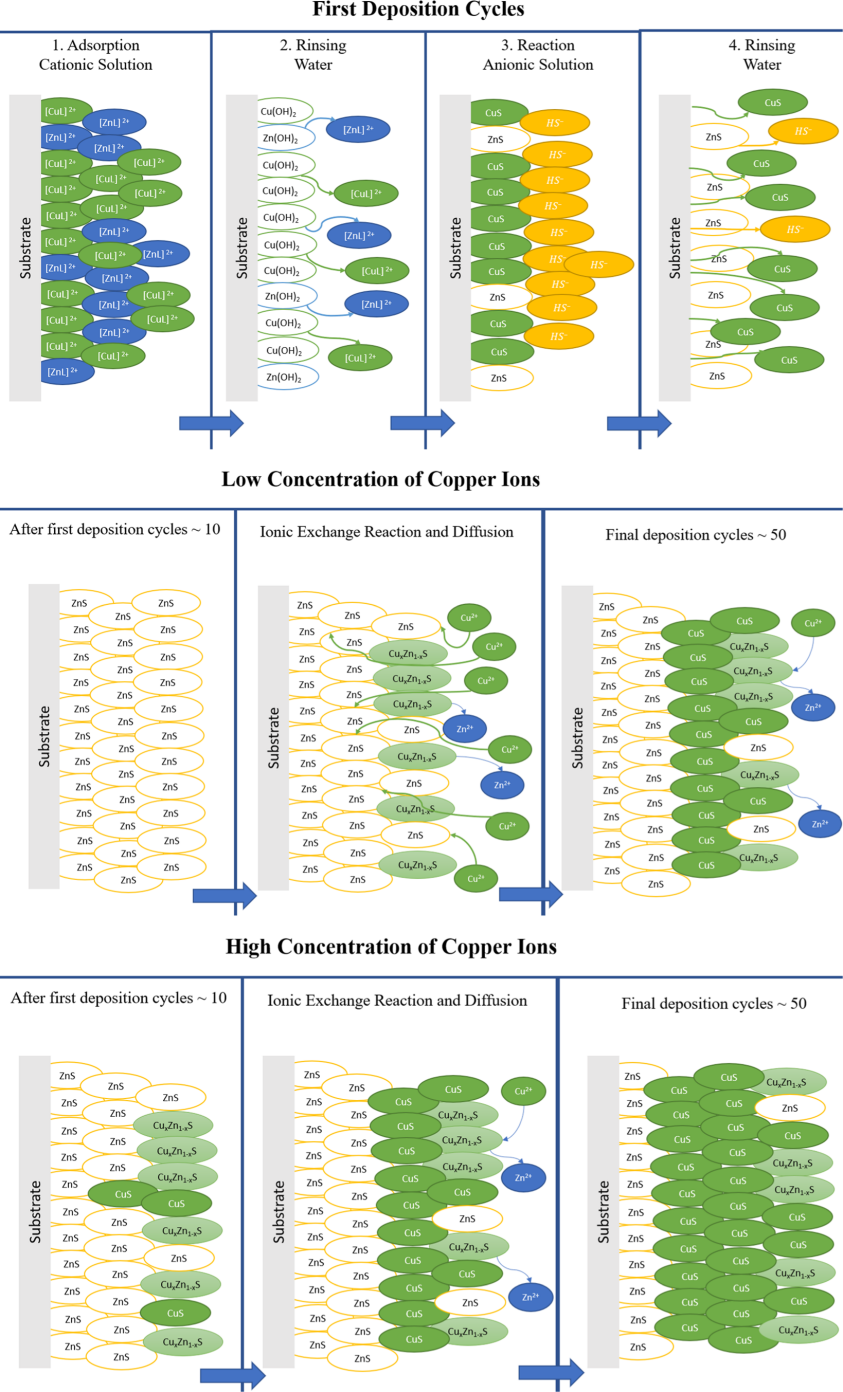
Schematic representation of the formation mechanism that describes the deposition of the Cu_*x*_Zn_1–*x*_S thin films. (L represents the possible complexed ions that could be formed with TEA, ammonium, or hydroxides present in the reaction solution.)

We propose that the latter description occurs during cationic solution preparation. Then, the thin-film deposition initiates after the glass substrate is introduced into the cationic solution, where the complexed aqueous ions are adsorbed to the glass surface via the negative charges present in the surface of the glass substrate.^74−76^ At this point, with the immersion of the substrate in the cationic solution, we have a tightly adsorbed layer of complexed ions on the surface of the substrate, accompanied by loosely adsorbed complexed ions,^77^ as shown in the first schematic (top left corner) of Figure 8, as is expected for the first step of the SILAR methodology. Collectively, the complexed ions that can be formed with ammonium and TEA are termed [ZnL]^2+^ and [CuL]^2+^.

Next, regarding the rinsing step in Figure 8, the substrate with adsorbed complexed ions is transferred to the first rinsing step, where foreign particulates and weakly adsorbed ions are removed from the substrate surface. Depending on the nature of the ionic compound that is tightly adsorbed on the surface, in this rinsing step exists the possibility for the formation of the metallic hydroxides—typical behavior of materials synthesized by solution methods—which in this case would be influenced by the Zn/Cu ratio of the initial cationic solution.

The following step in the thin-film deposition concerns the reaction, where sulfur ions (from the hydrolysis of the anionic solution) are available to react with the surface filled with metal hydroxides, producing metallic sulfur nuclei at the surface. The reaction solution containing Na_2_S provides sulfur ions like this
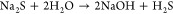
7
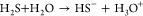
8

The initial SILAR cycle is completed with the second rinsing; up to this point, the surface of the substrate should maintain nuclei of ZnS and CuS as the first layer of the material; however, it is known that the deposition of copper sulfide has difficulties in ensuring its formation through SILAR due to the low or null adsorption/reaction of the ionic species of interest on the glass, for which a seed layer is needed.^21^ Thus, it is suggested that after the second rinse, the surface substrate is primarily covered with ZnS nuclei. Then, the formation of the Cu_*x*_Zn_1–*x*_S thin films requires the initial deposition of the seed layer which in this case is ZnS (top right corner, Figure 8) that occurs during the first deposition cycles (middle left, Figure 8).

After the first deposition cycles and the required ZnS seed layer are formed (around 10 cycles, determined by preliminary work), the copper ions are introduced into the film via an exchange reaction
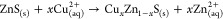
9

At low concentrations of copper ions in the cationic solution, there will be the formation of the metastable Cu_*x*_Zn_1–*x*_S (center, Figure 8), and as the concentration increases (around the 1.00 mM Cu^2+^ sample and above), the CuS covellite appears as the predominant material, which is evidenced by the XPS analysis. It is important to mention that during the exchange reaction, copper reduces from Cu^2+^ to Cu^+^ (shown by the characteristic Cu 2p photoemission spectra), and assuming a charge neutrality scheme, the oxidation state of sulfur is oxidized forming different oxidation states, which are verified by the presence of various peaks in the photoemission spectra of S 2p. Therefore, from the previous result, we have that the oxidation state of sulfur is a function of the degree of incorporation of copper
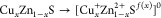
10

Through the Cu_*x*_Zn_1–*x*_S formed on the surface, copper ions diffuse toward the interior of the film gradually increasing the concentration of copper in the films up to the formation of the CuS covellite around the last deposition cycles (middle right, Figure 8). Obeying the following reactions
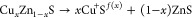
11
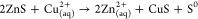
12

Moreover, the formation of CuS covellite for the high copper ion concentration may also obey the previous description, but in this case, the thin-film deposition is completely dominated by the growth of covellite CuS, as seen in the bottom section of Figure 8.

It is important to note that in the previous reaction, the chemical state of sulfur can take the form of S^2–^, S^–^, or any polysulfide unit within the covellite structure of the S_*n*_^2–^ type. Also, as shown in eq 12, elemental sulfur can also be formed by this mechanism, the presence of which was observed in the S 2p photoemission spectra.

To summarize the results, we finally present the possible general steps and reactions that take place during the SILAR thin-film deposition, which are as follows.

1. Adsorption and the successive reaction of ZnS.

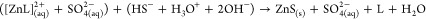

2. Ionic exchange and diffusion of Cu^2+^ forming metastable sulfide, where sulfur adopts oxidation states such as S^2–^, S^–^, and S_*n*_^2–^.

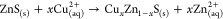

3. Reduction of copper ions to Cu^+^ and oxidation of S^2–^.

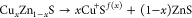

4. Covellite formation from a metastable sulfide, where elemental sulfur is also formed.

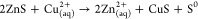

5. Adsorption and the successive reaction of ZnS and CuS on metallic sulfides in the final SILAR cycles.

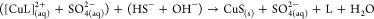


Within this proposed description, it was shown that through the SILAR methodology followed, it is possible to obtain the formation of the ZnS film, the Cu_*x*_Zn_1–*x*_S films with low concentrations of copper ions, and the formation of CuS covellite for the high concentration of copper ions.

## 4 Conclusions

This study presents a SILAR methodology that enables the thin film deposition of Cu_*x*_Zn_1–*x*_S across a wide range of compositions, starting from pure ZnS up to a predominant covellite CuS thin film. All the synthesized Cu_*x*_Zn_1–*x*_S thin films exhibit strong adherence to the glass substrate, which appears to be influenced by the initial formation of a ZnS seed layer upon which copper sulfides grow. We have demonstrated that the thickness, crystallinity, optical, and electrical properties are greatly influenced by the ionic concentration of the cationic solution during the SILAR processing, which in turn has direct repercussions on the copper content in the Cu_*x*_Zn_1–*x*_S thin films. The results are promising because, via careful preparation of the cationic solution, it is possible to tune the optoelectronic properties of the films. The careful analysis of the photoemission spectra provides interesting insights into the chemical nature of the films, mainly that the formation mechanism of the Cu_*x*_Zn_1–*x*_S thin films does not correspond to the standard SILAR mechanism but it is governed by ionic exchange reactions and diffusion processes dependent on the concentration of copper ions in the cationic solution. These results show that the thin films have a gradient-like composition depth profile where ZnS, Cu_*x*_Zn_1–*x*_S, and CuS coexist in the same film. Although there was no direct evidence for a stable ternary Cu_*x*_Zn_1–*x*_S phase, the photoelectron spectra do show complex electronic structures that could not be attributed solely to ZnS or CuS, highlighting the importance of more detailed studies of Cu_*x*_Zn_1–*x*_S thin films. We have also provided a complete chemical composition assessment of the films and observed that the films have an excess of sulfur that exceeds the expected stoichiometry. Moreover, including elemental sulfur coming from the ionic exchange reactions, several oxidation states for sulfur have been identified (that need further theoretical confirmation), which appear to be influenced by the amount of the predominant copper oxidation state. The careful analysis of the S 2p photoelectron spectra happens to be paramount for a more in-depth understanding of the relationship between structure and optoelectronic properties. It is worth noting that the stability of the films remains a factor that could influence the transparency and overall performance of the films because our results show that oxides do form at the surface of the films, and this could open an investigation possibility for the role of oxygen in Cu_*x*_Zn_1–*x*_S thin films. Overall, the Cu_*x*_Zn_1–*x*_S thin films synthesized in the present work show optoelectronic properties that may be suitable for photovoltaic devices, but a solid correlation between structure, optoelectronic properties, and photoelectron spectra is still needed for a clearer understanding and application of Cu_*x*_Zn_1–*x*_S, mainly detailed analysis around the optimal chemical composition of the thin films.
